# CONSIDERING HOSPITAL CLOSURES IN ALABAMA: NATIONAL POLICY IMPLICATIONS

**DOI:** 10.1093/geroni/igad104.2333

**Published:** 2023-12-21

**Authors:** Avani Shah, Joseph Swain, Hailey Lewy

**Affiliations:** University of Alabama, Tuscaloosa, Alabama, United States; Arkansas Tech University, Russellville, Arkansas, United States; University of Alabama, Tuscaloosa, Alabama, United States

## Abstract

Hospital closures have increased over the past 10 years nationally, particularly in rural areas and states that did not receive Medicaid expansion funds (American Hospital Association). These closures can impact older adults health care, employment opportunities, and mortality rates. Recently, the Alabama Hospital Association reported that 12 hospitals are at risk of closing. For this study, we evaluated the locations of hospital closures since 2009 in Alabama to understand gaps geographically in healthcare. We obtained information about current hospitals through Alabama Department of Public Health (ADPH). We searched for news articles to obtain a list of hospitals, which closed between 2009 and 2020. Next, we obtained data from the ADPH on the mortality rates by county and year. We also obtained information about the finances of hospitals in Alabama and classified hospitals with 20% losses as potentially at risk. Using GIS, we mapped areas of hospital closures and at risk hospitals as well as the location of current hospitals as of 2020. We chose to average mortality rates from 2009 to 2019 (pre-COVID) and map these by county. Since 2009, Alabama has closed 13 hospitals: with two reopening at a later date. Based on GIS, we found potential health care gaps in several rural areas and in Southwest Alabama, or the Black Belt area, which has a high rate of poverty, and poor health outcomes. The mean mortality rate for rural counties was significantly higher than urban populations between 2009-2019 t(54)=4.8, p<.0001. Policy implications will be discussed.

